# The Software Ontology (SWO): a resource for reproducibility in biomedical data analysis, curation and digital preservation

**DOI:** 10.1186/2041-1480-5-25

**Published:** 2014-06-02

**Authors:** James Malone, Andy Brown, Allyson L Lister, Jon Ison, Duncan Hull, Helen Parkinson, Robert Stevens

**Affiliations:** 1 EMBL-EBI, Wellcome Trust Genome Campus, Cambridge, CB10 1SD, UK; 2 School of Computer Science, University of Manchester, Oxford Road, Manchester, M13 9PL, UK

## Abstract

**Motivation:**

Biomedical ontologists to date have concentrated on ontological descriptions of biomedical entities such as gene products and their attributes, phenotypes and so on. Recently, effort has diversified to descriptions of the laboratory investigations by which these entities were produced. However, much biological insight is gained from the analysis of the data produced from these investigations, and there is a lack of adequate descriptions of the wide range of software that are central to bioinformatics. We need to describe how data are analyzed for discovery, audit trails, provenance and reproducibility.

**Results:**

The Software Ontology (SWO) is a description of software used to store, manage and analyze data. Input to the SWO has come from beyond the life sciences, but its main focus is the life sciences. We used agile techniques to gather input for the SWO and keep engagement with our users. The result is an ontology that meets the needs of a broad range of users by describing software, its information processing tasks, data inputs and outputs, data formats versions and so on. Recently, the SWO has incorporated EDAM, a vocabulary for describing data and related concepts in bioinformatics. The SWO is currently being used to describe software used in multiple biomedical applications.

**Conclusion:**

The SWO is another element of the biomedical ontology landscape that is necessary for the description of biomedical entities and how they were discovered. An ontology of software used to analyze data produced by investigations in the life sciences can be made in such a way that it covers the important features requested and prioritized by its users. The SWO thus fits into the landscape of biomedical ontologies and is produced using techniques designed to keep it in line with user’s needs.

**Availability:**

The Software Ontology is available under an Apache 2.0 license at http://theswo.sourceforge.net/; the Software Ontology blog can be read at http://softwareontology.wordpress.com.

## Background

We report on the Software Ontology (SWO) [1,2], an ontology for describing the software used within computational biology, which includes bioinformatics resources and any software tools used in the preparation and maintenance of data. Development of the SWO is motivated by the growing interest in the recording and reproducibility of biomedical investigations [3,4]. Reproducibility is as important for computational investigations of data as it is for investigations in the ‘wet’ laboratory [5,6]. In order to understand research results presented from data analysis investigations or perform new analyses based on these results, it is important to know whence the data came, how they were analysed and with what tools. In a recent Science paper, Peng [7] suggested that making research that uses computational methods reproducible requires much greater attention to detailing the software as part of the experimental process. Gentleman *et al*[5] state the need for reproducibility by combining analysis code with the data; e.g., using BioConductor packages to analyze MicroArray data. However, for reproducibility, the version of the BioConductor packages, R and any associated software that may have an influence on the outputs would need to be known - and even the hardware upon which it was run, as all of these can have an influence on the results obtained.

The growing use of workflows as a means of analyzing biological data [8-10] and as a means of recording and exchanging method [11] has provided one avenue for the recording of method. There has also been a move to automatically describe the provenance of computations (including the actual run of a workflow), and ontologies have been provided to support this recording [12]. An ontology such as the SWO provides the vocabulary and identifiers for the software aspects of such automatically recorded provenance.

As well as the reproducibility angle, describing software and the data it consumes and produces is important for search for software and construction of applications and workflows. Registries such as BioCatalogue [13] describe Web services used in bioinformatics according to data consumed and produced, and the functional units of the services involved and so on. These semantic descriptions can be then used for search and retrieval. Similarly, automated or semi-automated workflow construction depends on descriptions of the services [14].

An ontology of software can have impact in all of these areas by providing the means to describe software used, the data consumed and produced, its versions and so on. The scope of SWO is thus broad; it needs to cover not only bioinformatics, but any tools used in the management, analysis and presentation of biological data. *Prima facie*, the SWO needs to cover, but is not limited to, this range of software, and descriptions of its objectives (for what it is used), the data it consumes and produces, the algorithms it implements to achieve these objectives, its version, and some aspects of its project details. This software would include, for example, spreadsheets, word-processors, databases, as well as the bespoke desktop and services on the Web used by bioinformaticians within computational biology. A rich description of such a broad range of software used in life science investigations implies a similarly broad scope for an ontology of software. To date, however, attempts to produce such an ontology have not been convincing, although some promising efforts have been made: 

•DOAP (Description Of A Project) [15] describes a software project (home page, developers, language, etc.), rather than the software itself. There is an overlap in scope—the home page and developers are features that the SWO will need, but the SWO aims to model the software artifact itself, not the project.

•OWL-S [16] is a domain neutral and general mechanism for describing Web services, such that they can be discovered, composed and invoked. Of its three aspects, OWL-S’s profiling mechanism comes within the scope of the SWO, but the ‘grounding’ for the automatic invocation of a Web service is outside the SWO’s scope. In addition, its focus on only Web services precludes its model from our use. WSMO [17] (Web Services Modeling Ontology) has a similar remit and again is not suitable for the SWO’s aims.

•Ontologies of data mining tools cover much of the same ground as the SWO, but are obviously restricted to data mining. As data mining tools are used within bioinformatics and computational biology, we aim to include such descriptions into the SWO, and we incorporate portions of DMOP [18] into the SWO.

•The Ontology of Biomedical Investigations (OBI) [19] has a broad remit of enabling scientists to accurately and precisely describe how a biomedical investigation was planned and performed, including devices, samples, sample preparation, etc. As its remit is broad, it has also spun out ontologies such as the Information Artifact Ontology (IAO) [20] to allow descriptions of information such as digital documents associated with an investigation. Descriptions of software used in an investigation could fit within OBI’s remit, but the SWO has been deliberately kept separate as its scope is greater than biomedical investigations themselves. However, the SWO is built to be broadly inline with OBI and IAO.

•EDAM [21] is a vocabulary for describing data related concepts in bioinformatics including types of data, identifiers, formats, operations and ’topics’ of broad biological areas. EDAM is limited to these concepts and as such does not cover *software* and related software items like algorithms and licenses.

•The SWO aims at a wider scope of describing any software used in the pursuit of computational biology; this includes spreadsheets, database systems, XML parsers, ontology development environments and the like. EDAM is now a subset of the SWO and has been integrated into the SWO.

•The Bioinformatics Resource Ontology (BRO) [22] has a similar remit to EDAM—resource description and discovery. However, it models resources very broadly, capturing concepts concerning research infrastructures, people, funding etc. It also conflates various aspects of software, such as algorithm and implementation.

•The ^my^Grid Ontology [23], though focusing on bioinformatics software resources, makes many of the same distinctions as the SWO, but has not been actively maintained for a long-time. EDAM covers the concepts included in ^my^Grid and, with the SWO, supersedes and replaces the ^my^Grid ontology.

Bio-ontologies now cover a broad range of life science entities from biological sequences (SO) [24] to the functional attributes of gene products (GO) [25], and from cells (CTO) [26] to gross anatomy (Uberon) [27] and phenotype (PATO) [28]. We now also have ontologies describing small molecules and their roles that participate in many biological processes (ChEBI) [29]. Added to this are descriptions of biomedical investigations such as the OBI [19], the Experimental Factor Ontology (EFO) [30] and the BioAssay Ontology [31]. The SWO fits neatly and independently into this ontology landscape in its role as an ontology concerned with the description of resources used in the investigation of biomedical phenomena, rather than the biomedical phenomena themselves.

### User stories

The principal use for the SWO is in the description of resources used in storing, managing and analyzing data. Our SWO workshops produced a broad range of what in agile development are termed ‘user stories’ for the SWO [32] and we highlight a few here: 

•Describing the software used in the analysis of gene expression data. With the development of next-generation high throughput sequencing techniques, a slew of new software packages for analyzing this data has emerged. Understanding how results have been produced requires knowledge of the software used. This is an important consideration for the European Bioinformatics Institute (EBI), which hosts such data resources as ArrayExpress [33] where describing how data were analysed is important.

•The eagle-I project [34] concerns the collection of descriptions of biomedical resources and services available at various sites in North America such that consumers can find those relevant to their work. The eagle-I consortium require descriptions of data types and formats, software names, programming languages and licensing information to describe these resources.

•The European consortium under the BioMedBridges project [35] wish to collect descriptions of Web services and software tools used both within the consortium and more widely in Europe in a *Tool and Service Registry*[36]. They wish to answer user questions about software attributes such as data types and formats, licenses, and developer and source code information and function. Queries require structure for faceting such that higher-level categories in the ontology can be used to ‘drill down’ to subsets of interest.

•The British Library and UK National Archives require software descriptions to assist in the curation of digital artifacts for preservation purposes. Data curated using specific versions of a piece of software can produce varied results, so being able to describe attributes such as version and data formats are of great importance.

## Materials and method

The Software Ontology has adapted agile software engineering methods into the ontology engineering process [1]. Agile methods offer a number of principles that aim to keep users involved in the process of developing software and enable rapid response to changing requirements whilst also building in consistent quality control checks [32,37]. Specifically, the SWO project focused on the following agile principles [38] and adapted them to ontology development: 

•The SWO’s users, domain experts, and ontology engineers are all active contributors throughout the process;

•Close engagement with users meant the introduction of requirements gathering and ontology modelling sessions as iterative activities, each iteration (sprint) resulting in a new increment;

•Acknowledgment that requirements can evolve and priorities can change throughout the engineering lifecycle;

•Encouragement of self-organised and cross-functional teams of developers;

•Testing is an integral part of development and happens all the time;

•Provision of regular and frequent builds to the participants for discussion, testing and refinement (and ultimately agreement).

Applying these principles requires a number of events to take place in order to deliver information to other events in a cyclic manner, though events can be run in parallel. The agile ontology engineering method can be summarised as follows: 

•**Requirements gathering.** Requirements are captured from stakeholders by identifying key areas of interest, eliciting competency questions [39] and desirable features of the ontology. Activities are driven by user stories, in the form of competency questions, and card sorting exercises.

•**Requirements prioritisation.** Prioritisation of requirements has two components, both of which are adapted from agile engineering techniques. The first is the estimation of the complexity of implementing a particular requirement using ‘planning poker’ [32]. The second component allows participants to collectively rank requirements by ‘bidding’ on individual requirements of most interest to their needs based on the ‘Buy a Feature’ method [40].

•**Implementation of the ‘Top’ requirements.** The implementation of the ontology focuses on the prioritised requirements that were *bought* from the previous event. Separating the ontology components into modules allows concurrent development to occur from co-located or distributed developers. Additional content is gathered from stakeholders using methods such as template completion via tools, such as Populous [41] - a tool for creating ontologies using a spreadsheet like interface, which are well suited to large-scale concept collection.

•**Evaluation of product.** At the end of each iteration, the ontology is evaluated against competency questions [39]. Defined classes based on competency questions act as queries within the ontology and are used to demonstrate delivery of a particular feature to stakeholders.

The SWO project conducted three face-to-face workshops between 2011-2012 (see [42] for details), during which the method outlined above was applied [1]. The first workshop (WS1) was used primarily to gather requirements and potential content, since there was no ontology to evaluate at that point. The second (WS2) and third (WS3) workshops took place four months and 12 months later and were used to both evaluate form and content, as well as to generate new content for the SWO. There were 18 participants in WS1, 14 in WS2 and 17 in WS3. Seven of these participants attended all three workshops. Participants represented a user base under the broad heading of ’digital curation and preservation’, with more specific areas including archiving organizations, software sustainability, library services, astronomy, life science and pharmaceutical research.

### Spreadsheets and populous

Throughout the project, spreadsheets created using the Populous tool [41] were used to collect specific software descriptions from the community. Populous is a tool that allows cell values to be connected to ontology parts such that each row becomes a description for an ontology class following a specified template. In this way, members of the community did not have to learn new technology or ontology languages to contribute directly to the ontology; instead they simply worked in a familiar spreadsheet environment.

### Testing competency questions via DL queries

The testing component of the method concerns the use of competency questions phrased as description logic axioms executed as queries (DL queries). An ontology in OWL should be able to satisfy competency questions precisely and this can be tested using the description logic aspects of the language.

In the testing phase a DL query is formulated which represents a question of interest, e.g. which software can take as input image data in the JPG format images. If the DL query is not producing the desired results then the ontology needs further refinement and a further iteration occurs. Testing using DL queries in this way is a ‘test after’ [43] approach since test driven approaches require a test to be written before the encoding. This is not suitable for ontology development in most current environments since writing a DL query as a test to be executed before development requires testing infrastructure that, as yet, does not exist in most environments.

## Results

### What should be modelled in the SWO?

WS1 resulted in a set of requirements that the ontology was required to match; these were sorted into 15 groups of features, each group’s label became a *feature* for modelling in the ontology. In addition, there were 91 competency questions aligned to these features (see Table 1 for the feature groups and [44] for the competency question groups). For instance, the group *Function* contained sticky notes containing *can the software perform XML editing?* and *can the software be used for word processing?* It is worth noting that a question could also fall into multiple groups, for instance *can the software perform XML editing?* falls into both *Function* and, by implication, *Data/format* feature groups since the software would need to be able to parse XML. That all of the competency questions could be aligned to a feature group, and conversely that each feature group contained competency questions, provided a validation of the process, since an orphaned question might suggest a missing category or an empty feature group.

**Table 1:** The feature groups identified by the workshop participants

**Feature**	**Definition**	**Bought?**	**Example competency question**
Software	The software itself	Yes	What is the name of the software?
Data	Data that the software consumes and produces	Yes	Will it render a gif format image?
Function	The task the software is used to do, sometimes called objective	Yes	Does this software provide XML editing?
Algorithm	The specific instructions as part of software to perform a given task	No	What is the normalization algorithm used in this software?
Configure parameters	Parameters required to run the software; settings	No	What are setting needed to run this analysis?
Life cycle	Stage of maturity of a piece of software	No	Does the software meet the ISO-4 standard?
Version	The version information	Yes	What is the latest version of this software?
Supplier	Developer and/or maintainer of software	Yes	Who developed this software?
Dependencies	Other pieces of software or libraries required to run it	No	What are the dependencies for using OWL-API?
Interface	Modes of interaction with the software	Yes	Is there a Web API for Blast?
Source code location	URL or otherwise of source code	Yes	Where can I get the code?
Cost of ownership	Cost to purchase but also to run	No	Is it free?
Platform	Which platform is required to run software	No	Will the software run on Ubuntu?
License	What license and usage restrictions exist for a given software	Yes	What software can I use for my task which is under the Apache 2 license?
Architecture	Architectural structure of the software, such as peer-to-peer	No	Is the software client-server?

Those features that were ‘bought’ were then prioritised for inclusion in the ontology.

The list of features for the SWO gained from the workshops are shown in Table 1 along with whether or not they were ‘bought’, i.e. were prioritised in the user prioritisation sessions. From a modelling perspective, bought features were a combination of both simple concepts and more complex components; some features were deemed important but too costly to model in a way suited to customers’ needs, such as modelling the hardware upon which software is run. One interesting result of the prioritisation event is that the users initially suggested that some features, such as *algorithm*, were ranked highly, but following effort estimation suggesting this was very costly to represent, the feature was not bought. Some features which were discussed as important remained so after prioritisation and were duly bought, such as *data* and *function*.

In a second prioritisation event, the exercise was repeated. The algorithm component of software (originally not prioritised) was considered more important than had previously been determined and was added to the list of features. This became apparent after the initial examples failed to answer some of the competency questions regarding software that implements a given algorithm. Since there was a small amount of additional extra effort available, *algorithm* was included in some descriptions of software added more recently to the SWO.

### The ontology

**Figure 1:**
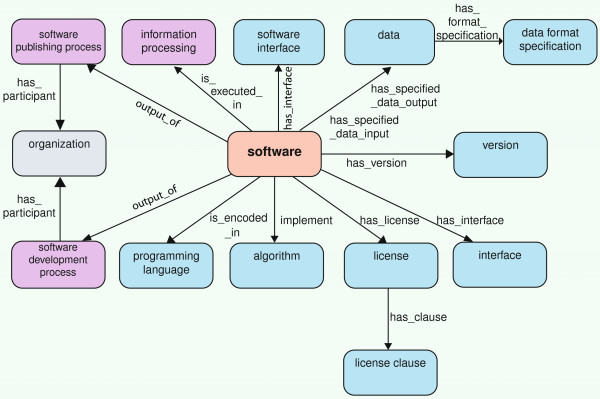
The SWO’s ‘schema’.

**Figure 2:**
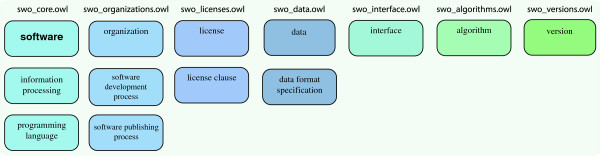
The SWO ’s ontology consists of several modules which are used to compose software descriptions.

The ontology was authored in the Web Ontology Language (OWL) [45] using the ‘schema’ shown in Figure 1 as a guide for the top-level distinctions made in describing software. As of Release 1.1 in December 2013, the SWO contained 3 777 classes, 50 object properties, 5 data properties and 114 individuals. Table 2 shows the number of classes under each major division in the SWO. Initially, addition of software used in bioinformatics to the SWO was driven by the needs of the ontology’s authors and client projects. Latterly, however, a more systematic approach has been adopted; we are using results of a survey of Genome Biology and BMC Bioinformatics with BioNERDS [46], a named entity recogniser for bioinformatics software and databases. This survey provided a list of software and databases ranked by the number of documents in which those resources were mentioned. We took the top 50 resources and removed the databases and any obviously spurious entries to leave only software (database management systems such as mySQL are software, but for the SWO a reference to database content, such as SWISS-PROT, does not count as software). Genome Biology gave 27 software names, and BMC Bioinformatics 25 names out of the top 50 resources in each case. These correlate to 47.5% of the total document level mentions within the top 50 in Genome Biology, and 53.7% in BMC Bioinformatics. In this way we expect to be able to make the SWO cover the main software used in bioinformatics and computational biology (the list of software is available in the supplementary data).The SWO is separated into discrete ontology modules that are combined to produce software descriptions. Separating the different aspects of software in this way allows for both concurrent development and reuse of those components useful for other projects, for instance the ‘organizations’ module for an ontology describing biomedical instruments and ‘license’ module for an ontology of literature. Figure 2 illustrates the different OWL module files and which components of the SWO they contain.

**Table 2:** The number of classes or individuals under each major division in the SWO; these are things that describe domain content, rather than ontology ‘infra-structure

**SWO division**	**Number**	**Example classes or individuals**
Software	512	Blast, Excel, Endnote, Clustal
Data	1168	heatmap, sequence alignment(protein), 2D PAGE image
Data Format	434	XLS, RDF-XML, BAM, JPEG
Information processing	608	Phylogenetic tree construction,spreadsheet editing, ontologyengineering,protein structure analysis
Algorithm	159	ANOVA, Chi-square, t-test
Organization	78	Agilent Technologies, AdobeSystems, Bioconductor, SASInstitute Inc.
Programming language	46	C++, Java, MATLAB language, Ruby
Software license	30	Apache license v2, GNU GPL, MITLicense

For describing the ontology we use the following font conventions: classes and *object properties*. The SWO is axiomatised as follows; the class Software is naturally the focus of attention. A class of software may be described in terms of the data it takes as input, the data it produces as output, the objective or processing task it is designed to meet, licensing restrictions that apply to using the software (and so on). Few of these properties are universally true of software (there is software that, at the granularity at which the SWO is represented) takes no data as an input), so using restrictions to represent these notions is not desirable. The only restriction on Software is that it is executed in some process. A typical piece of software would be described as follows:

•A property *has specified data input* links a software to its input data while *has specified data output* similarly links software to its output data. the ‘specified’ part of these property names seeks to capture the choice inherent in, for instance, data inputs by enabling statements such as ‘software x is specified to be able to take data types p, q and r as inputs’, without saying each and every instance of software actually does so. The tree of data is presently fairly flat, with some structure separating image data from much of the other data.

•Data format is separated from the data itself and is related via a property *has format specification* which can be used to specify that the data has a certain syntax, such as XML or SVG. In the SWO we make a distinction between data and the format of the data. These are easily conflated, but useful to pull apart as one type of data can be presented in many formats. Perhaps the easiest example is that of image data; here the data is the symbols or values that represent the meaning of the data; the format, however, is the syntax that governs the encoding and decoding of that data. Thus data are the symbols upon which a computer (typically via software) performs operations. the format specifies how the data are to be encoded. So, for images, image data is image data (some symbols), but an image file could be encoded in PNG, JPEG, PDF, etc, and, in some cases, inter-converted, preserving the image data itself.

•The algorithm section of the SWO captures algorithms which a piece of software *implements*.

•The property *is executed in* links a software to the information processing class in which it is executed. Information processing can be seen here as the task for which the software is being used to help accomplish. For instance, differential expression analysis and ontology engineering.

•Software can have a version using the *has version* property. Versioning is complex and is discussed in more detail below.

•Licenses are also described in the SWO as types of software license. Software licenses are described in terms of license clause classes via the *has clause* property which capture specific licensing aspects such as how software can be used, redistributed, extended or modified.

•Interfaces to software, such as APIs and graphical user interfaces, are described in software interface and related to software by the property *has interface*.

•Software is encoded within a programming language and this is represented via the property *is encoded in* the programming language part of the ontology.

•Organizations involved in developing or publishing software are captured as individuals under the organization class and related to software as the *output of* either via the software development process or the software publishing process.

•The SWO also contains datatype properties for connecting, for instance documentation locations to software with *has documentation*, homepage for software *has website homepage* and a download URL with *has download location*.

If we consider the example of Microsoft Excel™ 2007 this is described in the SWO as follows: 

•Excel is specified to be able to take as input any data in various data formats such as the XLS spreadsheet format and the tab delimited format. 

•Similarly, Excel is also specified as inputting and outputting data in various data formats such as the XLS spreadsheet format or as a tab delimited format. 

•Excel 2007 is versioned as Excel Microsoft 2007.

•‘has version’ value ‘Microsoft 2007 version’

•It has a proprietary commercial license.

•‘has license’ some ‘Proprietary commercial software license’

•It has a graphical user interface.

•‘has interface’ some ‘Graphical user interface’

•Excel 2007 is both developed and published by Microsoft. 

•Excel can be used to edit spreadsheets.

•‘is executed in’ some ‘spreadsheet editing’

For Excel, the specified inputs and outputs are Data, as spreadsheets can have content of a more or less arbitrary type. The SWO has not attempted to represent all possible formats for software such as Excel. Instead those data formats that are necessary for the annotations and searches for the SWO’s use cases are prioritised. In line with many ontology projects, the SWO is largely driven by the needs of its users.

BLAST 2.2.26 is described as follows: 

•BLAST 2.2.26 can take as input, for example, DNA sequence data in FASTA format or in GenBank format. 

•It has several interfaces including a command line interface and web based.

•‘has interface’ some ‘command-line interface’‘has interface’ some ‘web user interface’

•This version is developed by the NIH. 

•Blast can be used to perform multiple sequence alignment. It can also be used to perform pairwise sequence alignment.

•‘is executed in’ some ‘multiple sequence alignment’‘is executed in’ some ‘pairwise sequence alignment’

•It can be downloaded from ftp://ftp.ncbi.nih.gov/blast/executables/

•‘has download location’ value ‘‘ftp://ftp.ncbi.nih.gov/blast/executables/’’

The SWO makes a distinction between an item of software and a software suite; this is MS Word 2010 as opposed to MS Office 2010 that is a bundle of several MS products including MS Word. A software suite is a piece of software in its own right, as it provides a thin wrapper around the bundled software—even if this is just for presentational reasons. The SWO describes MS Office by using the property *has part* to relate the software components. For example, MS Office 2001:

has part some ‘Microsoft Excel 2002’has part some ‘Microsoft Word 2001’ 

#### Software licences

**Figure 3:**
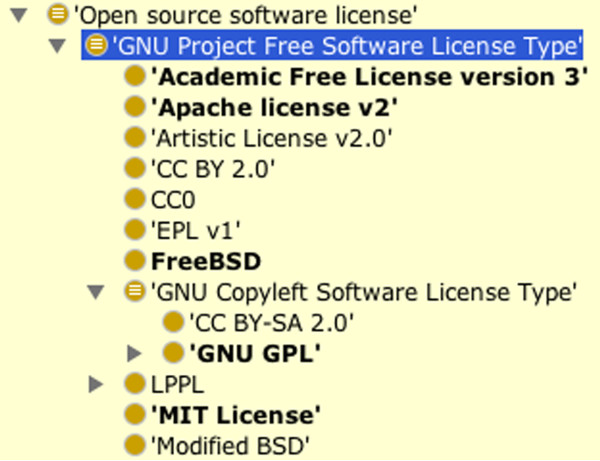
Inferring open source software licenses from the ontology.

Several competency questions focused on licensing issues such as ‘is the software open source’ or ‘available without restrictions on derivatives’. To capture this, software licenses were given parts, as mentioned above, which were ‘license clauses’. This way, a license can be described by attaching the relevant clause components which enables questions to be asked over these components. Figure 3 illustrates an example of a defined class that uses the same logic to infer types of software licenses that have clauses that indicate the software is open source. The highlighted class,’ GNU project Free Software License Type’ is described as follows: 

#### Software versions

The version name class is used to describe individuals which are a specific version name for a given piece of software. These versions are then related to the class of software with which they are associated using *has_version*. The version’s name is captured in the RDFS:label annotation of the given individual.

Competency questions for software versions required not only a record of what version name was attributed to a given software instance but also which versions preceded and proceeded a given piece of software. There are two forms of the question: Find all previous versions and find the version prior to the one in hand (and similarly for subsequent versions). This is a list of versions and we use the pattern described in [47]. The directly following and preceding version individuals are asserted via the properties *directly followed by* and *directly preceded by*. These properties have the super-properties *followed by* and *preceded by*, which are transitive (if A is preceded by B and b is preceded by C, then A is preceded by C). In OWL the sub-property implies the super-property, so the chain of transitive links is maintained automatically. This means that both forms of the competency question for versions can be answered. The variant of asking for the version *n* back in the chain would be answered with an expression like ‘directly preceded by’ some ‘directly preceded by’ ‘version x’ for the version two versions back in the list. In addition software which has a ‘dual’ licensing form (often for branding) can also be captured. In Manchester OWL this appears as follows: 

We can now perform the query by using the two transitive parent properties which will allow us to get, for example, all predecessors. Continuing the example, for versions of Microsoft Excel which came before this current version, in Manchester OWL: 

which when asked of the SWO returns the classes Microsoft Excel 2002 and Microsoft Excel 2003.

### Merging SWO and EDAM

The SWO has a broader scope of software than EDAM, but both broadly model software in the same way. As such, EDAM is a subset of the SWO, we have been merging EDAM into the SWO. Although much of EDAM is now merged into the SWO, there is still an ongoing process of refactoring to align these fully. Full details of the merge procedure can be found on the SWO blog [42]. The process to date can be summarised as follows: 

1. Modifications to the underlying annotations within EDAM were performed to align the structure of the ontologies more closely.

2. The native OBO format was converted to OWL.

3. High-level EDAM hierarchies were merged into the SWO structure.

#### Annotations and Conversion to OWL

A number of annotations were added to the EDAM ontology in preparation for its conversion to OWL and, ultimately, merging with the SWO. These included: 

•The addition of the *definition_editor* annotation property from EFO to all classes with definitions, providing authorship in a manner in line with the method already employed within the SWO.

•The addition of the EDAM idspace to all properties (and usages of those properties) within the OBO file as the automated conversion to OWL creates an incorrect OWL-based namespace,

•The addition of appropriate alternative annotations to the converted OWL files, as the OWLAPI 4.1 does not convert some annotations to OWL correctly,

•The automatic conversion of EDAM to OWL using Protege 4.1’s conversion feature, which makes use of the underlying OWLAPI. While the Protege 4.1 conversion process between OBO and OWL is straightforward, some manual changes were required [48]. The merge of EDAM into SWO and therefore into OWL will render this process unnecessary in the future.

#### Merging

There are four high-level EDAM terms: Data, Format, Operation and Topic. These terms and their hierarchies are in the process of being manually merged with the SWO. The initial stages of this have been previously described in [49]. In this process, each high-level EDAM term is compared against the SWO and either added as a subclass to an appropriate point (where no equivalent class exists) or formally axiomatised as equivalent to a pre-existing SWO class.

EDAM’s Format and Data have been fully merged, and can be found within the SWO as equivalent classes to data format specification and data, respectively. EDAM’s Topic class describes ‘broad domains or fields of interest’ and has no equivalent class within the SWO, and has been added without any modifications as a child of the SWO’s information class.

Initially it appeared that the EDAM Operation class would be a good match for the SWO Objective hierarchy. EDAM Operation describes tasks, such as ‘data annotation’ or ‘classification’ in much the same way as SWO objective. However EDAM’s Objective, defined as ‘information describing the intended outcome of running a process’, does not match the SWO’s Operation’s modelling of the whole process (inputs, outputs, process and outcome). As the definition of EDAM Operation class fitted better under process in the SWO, Operation has been merged with information processing (a child of process in the SWO) and the two classes have been axiomatised as being equivalent.

If EDAM’s Operation had been simply placed under process in the SWO, then the SWO Objective and newly-enhanced process hierarchies would have contained many similarities. For example, the EDAM sequence analysis class within Operation has many similarities with the SWO classes within Objective such as molecular sequence analysis. As such, Operation was first merged with the SWO information processing, then the SWO Objective hierarchy was refactored as part of the process hierarchy, and finally the Objective class itself was deprecated (for further details see [50]).

An additional issue arose with the EDAM class Parameter. Parameter was considered a class of data in EDAM whereas the contextual nature of whether or not something is a parameter would suggest it is a role in the SWO. The class metadata is a type of data in the SWO but in EDAM this is a type of report.

There is also a use of asserted multiple hierarchies in EDAM, for example BioXSD (format) class is an asserted subclass of five other classes; Alignment format (XML), Raw sequence format, Sequence feature annotation format, Sequence record format and XML. The SWO hierarchy enforces a single axis of asserted classification and multiple classifications are built by inference following a normalisation style approach [51]. EDAM did not have this strict constraint during its development, so in the merged SWO and EDAM asserted polyhierarchy exists, however, refactoring is ongoing to remove any remaining asserted polyhierarchy.

Some of this integration can be seen in Figure 3. The shared EDAM upper level classes with the SWO, such as data (Data in EDAM) and data format specification (Format in EDAM) can be seen here. Equivalence axioms were placed between classes where integration was clear (i.e. the ontologies referred to the same concept but with different URIs).

### The SWO’s polyhierarchy

The polyhierarchy produced and maintained in the SWO by this approach produces an ontology in which software is described along many dimensions. These dimensions are those captured in the properties and divisions within the SWO. As well as license and version above, software can also be classified along the other dimensions previously described, such as: 

•The data it takes as input:

•software and has specified data input some data

•The data it takes as output:

•software and has specified data output some data

•The process the software supports:

•software and is executed in some information processing

•The data format supported by the software

•software and has specified data input some (data and (has format some data format specification)) 

•and similarly for specified data outputs

•Algorithms implemented

• software and implement some algorithm

•Programming language

•software and is encoded in some programming language

•Developer of software

•output of some (software development process and (has participant value Microsoft))

These dimensions can be combined in arbitrary forms, e.g., Information processing task, inputs and outputs. Defined classes instantiating these classifications are not numerous within the SWO; instead these queries would be deployed at time of use from within software applications using the SWO.

### The SWO applied

#### BioMedBridges software registry

The Tools and Data Services Registry [36] is a catalogue of the prevalent bioinformatics tool and data resources, including the Web services, portals and applications used by scientists within the BioMedBridges research infrastructures. The registry, which is developed in a sustainable way by ELIXIR [52], requires a detailed description of software and resources. The vocabulary for this description is provided by the SWO and EDAM, and includes the type of software and software interface, topic (general scientific domain), function, types of input and output data, data formats, software maturity, supported platform, language, license and cost. The registry is built using a federated curation model in which software descriptions are harvested from key providers and other registries, working with these partners to ensure annotations are made at source. For example, the registry will include content from BioCatalogue [53], which will also be annotated using the SWO and EDAM.

#### eagle-I

The eagle-I network is a US$15 million NIH-funded project with the aim of facilitating biomedical research by creating a network of research resources repositories [34]. More than 50,000 resources which include biomedical data, software, databases and services - are listed and more are added every week. The Software Ontology plays an important role within the eagle-I’s application ontology which is used for indexing and searching these resources. This includes the discovery of resources based on data sets and formats, licenses and software function.

#### Gene Expression Atlas Data

The Gene Expression Atlas has produced an RDF representation [54] which describes summaries of whether or not a gene is differentially expressed given a particular condition, e.g. human liver. As part of these descriptions, SWO and EDAM classes are used to capture which software analysis packages were used to produce the summary information and to type data resources which link to this gene expression data, such as an Entrez Gene Database Reference. SWO was also applied to the RDF export of this data into the new EBI RDF Platform [55] wherein the statistical packages used to generate the results were typed with SWO classes enbling querying over the specific software.

### Evaluating the SWO

Our evaluation of the SWO took two forms: 

1. *Testing by competency questions*—Do we meet the tests as supplied by the competency questions set by our customers?

2. *Coverage*—Does it it contain terms required to annotate with?

As described above, the SWO has been used to describe software in several settings. The informal feedback from users browsing the ontology is that the SWO has the appropriate ‘shape’ and talks about the right features for customer’s tasks. The BMB project has, however, raised the issue of describing the platform upon which the software is capable of running or was run in a particular setting. This was raised as an important issue in the SWO workshops, but a complete description was deemed too costly to be ‘bought’. A similar missing feature is the cost of software; again, this was raised in the workshops, but was not a high enough priority to be bought. Cost covers many facets—there’s the monetary cost, but there is also the cost of use and maintenance. Monetary cost is relatively straight-forward to model, but the other costs are highly subjective. Our current thoughts are to use a rich description of licences to imply whether or not a software is ‘free to use’ and form slightly more complex axioms to cover the case when the software is free to a subset of users, for example ‘free to academics’ and such like. This latter modeling of cost is now being built into the latest versions of the SWO.

As well as the features described, the in-use evaluation naturally reveals a lack of content; the software that needs to be described is not present. As previously mentioned, as well as direct submissions from the community, the SWO has more recently been evaluating against the BioNERDS list of software mentions in biomedical literature and is looking to improve to 100% coverage of the top 200 within the next 6 months. The dynamic and fluid nature of software availability and development within the bioinformatics community is an ongoing issue and is not unique to the SWO. The SWO has reused, where possible, reference bio-ontologies such as the OBO Relation Ontology and Information Artifact Ontology and has consulted with various other ontology consortia on the model used to describe software. This has helped to populate some small areas of the ontology more quickly than others, though generally much of what is in the SWO does not exist within these reference ontologies, reinforcing the need for an ontology like the SWO.

Our on-going testing and ontologising to pass failed tests works as a tactic in ontology development. However, frameworks for doing this are only nascent. The processes used in the SWO aspire to follow similar methods to those used in the development of production ontologies such as the EFO at the EBI [30]. Here, continuous integration systems are used to test each commit of a version of an ontology such that potential bugs are caught early.

## Discussion and conclusions

An ontology of software is necessary for the description of the data that are now central to the pursuit of life science research. Just as we need ontologies to specify the biomedical entities that are discovered through our science, we also need a description of how those entities were discovered—both in the wet lab and the dry computational analysis of the data produced by those biomedical investigations. The SWO fits into this ontological landscape.

Descriptions of a software’s information processing tasks, the data it consumes and produces, together with the format of those data, are central to the SWO. In addition to the core areas, the SWO describes many peripheral but useful concepts including software developers and their organizational background as well as software versions, locations, and licensing. To create an ontology that is complete in any of these areas is ambitious. For instance, it is not feasible to describe the universe of softwares’ information processing tasks. Instead, the SWO takes the stance of doing what is necessary for the job in hand; our Agile approach should help in keeping the SWO fit for purpose. Nevertheless, the SWO’s conceptual framework seeks to be able to accommodate the changes necessary to keep it fit for purpose.

The work to integrate with EDAM has enriched the SWO with additional concepts in the areas of bioinformatics resources and Web services. In the context of wider biomedical investigations, the SWO with EDAM should play a significant role in annotating experimental protocols, alongside complementary ontologies such as OBI.

Biomedical ontologies typically focus on biological and medical entities which introduces its own levels of complexity, particularly placing knowledge into the context of evolution. Biomedical software faces different complexities; evolution is replaced with the diversities of human design and practice. It is clear that this variation introduces difficulties in making biomedical analyses both describable and reproducible but this requires more than just the appropriate ontologies to be available. There needs to be a paradigm shift towards both releasing all data associated with investigations and in describing the components in sufficient detail that they are understandable and reproducible. This issue only becomes more salient in the age of so called Big Data, lest we face the problems we already encountered when interpreting the current archive of Medium Data [56]. This requires a combination of elements including tooling, funding and the treatment of metadata as a first class citizen. An ontology of software will play an important role in achieving this aim.

The SWO has been developed under the Apache 2.0 open source license and is open to collaboration from external bodies. Already, several groups are making edits to the ontology and we hope to increase this number with additional members of the community. New user groups have recently emerged such as the new CLI-mate [57] tool and we intend to support these activities.

## Competing interests

The authors declare that they have no competing interests.

## Authors’ contributions

JM, AB, ALL, JI, DL, HP and RS contributed content to the SWO. JI is lead developer of EDAM. JM and RS managed the SWO project and organised user workshops. All authors read and approved the final manuscript.
